# Long‐acting reversible contraception: A route to reproductive justice or injustice

**DOI:** 10.1002/imhj.21801

**Published:** 2019-07-22

**Authors:** Marsha Kaitz, David Mankuta, Lihi Mankuta

**Affiliations:** ^1^ Department of Psychology Hebrew University Jerusalem Israel; ^2^ Department of Obstetrics and Gynecology Hadassah Hebrew University Hospital Jerusalem Israel; ^3^ Department of Medicine Faculty of Health Sciences Ben‐Gurion University of the Negev Beer‐Sheva Israel

**Keywords:** LARC, long‐acting reversible contraception, reproductive health, reproductive justice, pregnancy, unintended pregnancy, Contracepción Reversible de Larga Actuación (LARC), salud reproductiva, justicia reproductiva, embarazo, embarazo no intencional, Contraception à long terme et réversible (LARC), santé reproductive, justice reproductive, grossesse, grossesse involontaire, reversible Langzeitkontrazeptiva (Long Acting Reversible Contraception LARC), reproduktive Gesundheit, reproduktive Gerechtigkeit, Schwangerschaft, unbeabsichtigte Schwangerschaften, 長時間作用型可逆性避妊法 (LARC), 生殖の健康, 生殖の公平性, 妊娠, 意図しない妊娠, 長效可逆性避孕 (LARC), 生殖健康, 生殖公義, 懷孕, 意外懷孕, وسائل منع الحمل العاكسة طويلة المفعول (LARC), الصحة الإنجابية, العدالة الإنجابية, الحمل, الحمل غير المقصود

## Abstract

This article presents information on unintended pregnancies and the ongoing efforts of policy makers to promote long‐acting reversible contraception (LARC) to reduce the numbers of such pregnancies. Also discussed is the tension between the encouragement of LARC to promote the public's interests in achieving that goal versus the need to assure that all women can decide about their bodies and reproductive needs. Our discussion includes information, primarily from the United States, on (a) risks associated with unintended pregnancies, (b) LARC devices approved in the United States (copper intrauterine devices (IUDs), hormone IUDs, and implants), (c) public and social benefits of increasing the use of LARC, (d) disadvantages and barriers to using LARC, (e) dangers of promoting LARC in unjust ways, and (f) the meaning of reproductive justice and its connection to social justice. By sharing the information with the audience of this journal, we hope that it will be integrated into clinical work and research on mental health and development. We also hope that experts in those fields will become discussants in the conversation regarding women's reproductive health and social justice that is taking place in the United States and elsewhere.

## RESUMEN

Este artículo presenta información sobre embarazos no intencionales y el continuo esfuerzo de las autoridades para promover LARC (Contracepción Reversible de Larga Actuación) con el fin de reducir el número de tales embarazos. También se discute la tensión entre el aconsejar LARC para promover los intereses públicos de alcanzar esa meta vs. la necesidad de asegurar que todas las mujeres puedan ellas mismas decidir sobre sus cuerpos y necesidades reproductivas. Nuestra discusión incluye información, primariamente de los Estados Unidos (EUA), sobre: (1) riesgos asociados con embarazos no intencionales, (2) objetos de LARC aprobados en EUA (objetos intrauterinos de cobre ‐IUD‐, IUD de hormonas, e implantes), (3) los beneficios públicos y sociales de aumentar el uso de LARC, (4) desventajas y barreras que presenta el uso de LARC, (5) peligros de promover LARC de maneras injustas, y (6) el significado de la justicia reproductiva y su conexión con la justicia social. Al compartir la información con el público de esta revista especializada, esperamos que la misma sea integrada dentro del trabajo clínico y la investigación sobre salud y desarrollo mental. También esperamos que los expertos en esos campos de estudio participarán activamente en la conversación acerca de la salud reproductiva de las mujeres y la justicia social que se lleva a cabo en EUA y otros lugares.

## RÉSUMÉ

Cet article porte sur les grossesses involontaires et les efforts continus que font les responsables politiques pour promouvoir la contraception à long terme et réversible LARC (en anglais *Long Acting Reversible Contraception*) de façon à réduire le nombre de ces grossesses. Nous discutons aussi la tension entre l'encouragement de la LARC à promouvoir les intérêts publics pour arriver ce but et le besoin qui existe de s'assurer que toutes les femmes puissent décider d'elles‐mêmes ce qu'elles veulent faire avec leur propre corps et leurs besoins sexuels. Notre discussion inclut des renseignements, principalement des Etats‐Unis d'Amériques, sur: (1) les risques liés aux grossesses involontaires; (2) les dispositifs de contraception à long terme réversible approuvés aux Etats‐Unis d'Amérique (dispositifs intra‐utérins au cuivre (DIU), hormones DIU, et implants), (3) les avantages publics et sociaux qu'il y a à augmenter l'utilisation de la LARC, (4) les désavantages et les barrières à l'utilisation de la LARC, (5) les dangers de la promotion de la LARC de manières injustes, et (6) la signification de la justice reproductive et son lien à la justice sociale. En partageant ces informations avec les lecteurs de cette revue, nous espérons qu'elles seront intégrées dans le travail clinique et les recherches sur la santé mentale et le développement. Nous espérons aussi que les experts dans ces domaines pourront ainsi intervenir dans la conversation qui concerne la santé reproductive des femmes et la justice sociale qui se tient aux Etats‐Unis et ailleurs.

## ZUSAMMENFASSUNG

Dieser Artikel enthält Informationen über unbeabsichtigte Schwangerschaften und die laufenden Bemühungen der politischen Entscheidungsträger, reversible Langzeitkontrazeptiva (Long Acting Reversible Contraception; LARC) zu fördern, um die Zahl dieser Schwangerschaften zu reduzieren. Ebenfalls diskutiert wird die Spannung zwischen der Unterstützung der LARC, um die Interessen der Öffentlichkeit bei der Erreichung dieses Ziels zu fördern, und der Notwendigkeit, sicherzustellen, dass alle Frauen selbst über ihren Körper und ihre reproduktiven Bedürfnisse entscheiden können. Unsere Diskussion umfasst Informationen – hauptsächlich aus den Vereinigten Staaten (USA) – über: (1) Risiken im Zusammenhang mit unbeabsichtigten Schwangerschaften, (2) in den USA zugelassene LARC‐Methoden (Kupfer‐Instrauterinpessar (IUPs), Hormon‐IUPs und Implantate), (3) öffentliche und soziale Vorteile einer verstärkten Verwendung von LARC, (4) Nachteile und Barrieren bei der Verwendung von LARC, (5) Gefahren einer Förderung von LARC auf unrechtmäßige Art und Weise und (6) die Bedeutung der reproduktiven Gerechtigkeit und ihr Zusammenhang mit sozialer Gerechtigkeit. Indem wir die Informationen mit der Leserschaft dieser Zeitschrift teilen, hoffen wir auf die Integration der Informationen in die klinische Arbeit und Forschung zur psychischen Gesundheit und Entwicklung. Wir hoffen auch, dass Experten auf diesen Gebieten zu Gesprächspartnern in der Debatte über die reproduktive Gesundheit und soziale Gerechtigkeit von Frauen werden, die in den USA und anderorts stattfindet.

## 抄録

長時間作用型可逆的避妊法[LARC]: 生殖の公平あるいは不公平性への道筋

本論文では、意図しない妊娠の数を減らすために、LARC (長期作用型可逆的避妊法) を促進するための政策立案者の継続的な取り組みと、意図しない妊娠に関する情報を述べる。また、その目的を達成することへの大衆の関心を促進するために LARC を奨励することと、すべての女性が自分の身体や生殖の必要性について自分自身で決定できることを保証する必要性との間の緊張も議論している。

我々の議論は、主にアメリカ合衆国からの情報を含んでいる。それは、 (1) 意図しない妊娠に関連するリスク (2) 米国で承認されている LARC 器具 (銅付加子宮内避妊用具 (IUD) 、ホルモン IUD、およびインプラント) 3) LARC の利用を増やすことによる公的および社会的利益、 (4) LARC を利用することの不利益および障壁、 (5) LARC を不当な方法で推進することの危険性、および (6) 生殖の公平性の意味および社会的正義との関連。

この学会誌の読者と情報を共有することによって、精神保健及び発達に関する臨床と研究とに統合されることを願っている。また、これらの分野の専門家が、米国や他の地域で行われている女性の生殖の健康と社会正義に関する議論に加わってくれることを期待している。

## 摘要

長效可逆性避孕[LARC]: 生殖公義或不公義的途徑

本文介紹有關意外懷孕的信息以及政策制定者為促進 LARC (長效可逆性避孕) 而不斷努力, 以減少此類懷孕的數量。本文還討論了鼓勵 LARC 促進公眾實現該目標的利益, 與確保所有女性決定自己身體和生殖需求的必要性之間的緊張關係。我們的討論包括主要來自美國 (US) 的信息: (1) 與意外懷孕相關的風險, (2) 美國批准的 LARC 裝置 (銅宮內節育器 (IUD) , 激素宮內節育器和植入物) , (3) 增加使用 LARC 的公共和社會利益, (4) 使用 LARC 的不利因素和障礙, (5) 以不公正的方式促進 LARC 的危險, (6) 生殖公義的含義及其與社會公義的聯繫。通過與本期刊的讀者分享信息, 我們希望將信息納入心理健康與發展的臨床工作和研究。我們還希望這些領域的專家, 成為正在美國和其他地方發生的婦女生殖健康和社會正義對話的討論者。

## ملخص

وسائل منع الحمل العاكسة طويلة المفعول [LARC]: الطريق إلى العدالة الإنجابية أو العكس

تقدم هذه المقالة معلومات عن حالات الحمل غير المقصود والجهود الجارية التي يبذلها صانعو السياسات لتعزيز وسائل منع الحمل العاكسة طويلة المفعول (LARC) من أجل الحد من أعداد حالات الحمل هذه. ويناقش أيضا التناقض بين تشجيع وسائل منع الحمل لتعزيز مصالح الجمهور في تحقيق ذلك الهدف وبين الحاجة إلى ضمان أن تتمكن جميع النساء من اتخاذ قرار بشأن أجسادهن واحتياجاتهن الإنجابية بأنفسهن. تتضمن مناقشتنا معلومات، من الولايات المتحدة الأمريكية في المقام الأول، عن: (1) المخاطر المرتبطة بالحمل غير المقصود، (2) أجهزة LARC المعتمدة في الولايات المتحدة (اللولب النحاسي داخل الرحم(IUDs)، واللولب الهرموني ، والتركيبات)، (3) المنافع العامة والاجتماعية لنظام LARC، (4) المساوئ والحواجز التي تحول دون استخدام LARC، (5) مخاطر تعزيز LARC بطرق غير عادلة، و (6) معنى العدالة الإنجابية وارتباطها بالعدالة الاجتماعية. ومن خلال تبادل المعلومات مع جمهور هذه المجلة، نأمل أن يتم دمجها في العمل الاكلينيكي والبحوث المتعلقة بالصحة النفسية والنمو. ونأمل أيضا أن يصبح الخبراء في تلك المجالات مناقشين في الحوار المتعلق بالصحة الإنجابية للمرأة والعدالة الاجتماعية الذي يجري في الولايات المتحدة وأماكن أخرى.

## 1 INTRODUCTION

Nearly half of pregnancies in the United States are unintended, meaning that the mother did not want to become pregnant at that time or at all (Finer & Zolna, 2016). To reduce rates of unintended pregnancies, efforts are ongoing to promote the use of Long Acting Reversible Contraception (LARC), which is the most effective reversible contraception available today (American College of Obstetrics and Gynecology, 2017). Many of these promotions are targeted at marginalized women, including those who are poor, of color, or very young (Finer & Zolna, 2016).

Efforts to promote LARC make sense on a number of levels since pregnancies and births covered by Medicaid^1^ are huge expenditures, so preventing those pregnancies is beneficial to government from a financial perspective (Sonfield & Kost, 2015). In addition, unplanned pregnancies can have negative effects on women (described later), so it could seem morally responsible to offer them the most highly effective contraceptive options. However, for minority and women's advocacy groups, the policies that direct women toward certain contraceptive practices read as a reinvention of the American eugenics movement and other concerted efforts to limit pregnancies of poor women and those belonging to minorities (Asian Communities for Reproductive Justice, 2005; Black Mamas Matter Alliance, 2018; Ross/SisterSong Women of Color Reproductive Justice Collective, 2006/2011). As such, LARC policies can be easily interpreted as unethical because they can undermine women's reproductive rights and could serve as a means to keep poor and minority women from reproducing. Although endorsing LARC devices (LARCs) for women who choose them freely, the reproductive justice (RJ) movement calls for family planning services that support each woman in identifying her family planning priorities and adopting the method that best meets her current needs. Beyond this, the movement addresses and calls for the rights of women to access equal‐quality healthcare and live in healthy and safe environments (Gomez, Fuentes, & Allina, 2014).

The tension between these two perspectives is deep, public, and ongoing; issues at the heart of the debate are highly relevant to infant mental health (IMH) professionals because women's reproductive health and their right to choose the reproductive strategies that are good and appropriate for them and their family can impact women, families, children, and family relationships, and these are the core domains of IMH. In addition, knowledge about reproductive health and justice can broaden the perspective of IMH professionals, encourage their self‐exploration as to personal prejudices and biases, improve clinical practice, and offer ideas for new lines of research. Knowledge about RJ also could suggest avenues of cooperation and collaboration between persons who work on behalf of RJ, social justice, and IMH (discussed later).

This article aims to provide IMH professionals with information on the controversies surrounding LARC, incorporating both the positions in favor of promoting LARCs over other contraceptives and those advocating RJ. Since efforts to promote LARC are related to the high incidence rate of unintentional pregnancies in the United States (and elsewhere), this article begins with a review of recent literature on outcomes associated with those pregnancies, followed by caveats to reported findings. We then describe LARCs approved by the U.S. Federal Drug Administration (FDA), followed by a discussion of the advantages and disadvantages and barriers to using them. We then describe some of the current initiatives for increasing LARC use and the variety of ways that they can be encouraged in socially unjust ways. Finally, we discuss the connection between RJ and social justice and between RJ, social justice, and IMH.

## 2 DEFINITIONS, INCIDENCE, AND RISKS ASSOCIATED WITH UNINTENDED PREGNANCIES

To begin this section on unintended pregnancies, we define focal constructs and describe a few methodological and statistical issues that are limitations of research on the topic.

### 2.1 Definitions

Unintended pregnancies (also referred to as “unplanned”) are pregnancies that are reported to have been either unwanted (i.e., they occurred when no children, or no more children, were desired) or mistimed (i.e., they occurred earlier than desired). Measures of assessing pregnancy‐intentionality (PI) vary across research studies. Some tools ask respondents to choose the most fitting category on binary (e.g., planned vs. unplanned) or multipoint scales (e.g., wanted vs. unwanted vs. mistimed); other tools ask women about their feelings (e.g., happy or not) or about behaviors such as whether they were using contraception when they conceived or tried to have an abortion. In one study, children born as a result of a denied abortion (considered “unwanted”) were compared to children conceived and carried to term shortly after their mother had an abortion (considered “wanted”) (Foster et al., 2018).

Measures and research design of studies on PI have been critiqued in previous studies (e.g., Santelli et al., 2003); the predominant ones are listed here:

- Scales with few response options (e.g., planned vs. unplanned) do not capture the fine shadings of PI. Some measures do not discern pregnancies that were mistimed versus those unwanted, and most do not tap the emotional, cognitive, and behavioral aspects of PI, which do not necessarily concur with one another (Trussell, Vaughan, & Stanford, 1999).
- Reports of PI are usually retrospective and, therefore, prone to bias. Retrospective measures are particularly vulnerable to ex‐post revisions because women are reluctant to label an existing child as unwanted. This could lead to an underestimation of unplanned pregnancies. Prospective measures also may be inexact if respondents are unwilling to report socially undesirable preferences.
- Contextual and personal variables can be associated with both unplanned pregnancies and outcomes associated with them. Studies that do not account for this entanglement by design or statistical analysis may incorrectly attribute an outcome to unplanned pregnancies when, in fact, it is actually due to factors, including more limited opportunities and structural (e.g., education) disadvantages associated with poverty, that could increase both the likelihood of an unplanned pregnancy and the likelihood of certain outcomes (Barber, Yarger, & Gatny, 2015; Kane, Morgan, Harris, & Guilkey, 2013).
- Research in the field of reproductive health typically uses population‐/group‐level statistics as a proxy for individual variables. Consequently, these studies run the risk of statistical discrimination; that is, classifying a woman's risk based on epidemiologic data or previous clinical experiences, without consideration of her history, preferences, and priorities (Balsa, McGuire, & Meredith, 2005). Such discrimination can lead to profiling due to group membership (e.g., low income) without consideration of other personal factors that may be equally or more significant.

These caveats advise caution in interpreting the results from studies on PI and suggest that future research is needed to identify differences and similarities between subgroups and between individuals to appreciate their commonalities and diversity.

### 2.2 Incidence

The National Center for Health Statistics, Healthy People 2020, and the Guttmacher Institute are among the leading health metrics organizations that track the incidence of unintended pregnancies in the United States. The most recent data have shown that unintended pregnancies continue to represent a significant portion of the 6.1 million annual pregnancies in the nation (Guttmacher Institute, 2016c). In fact, 45% of all pregnancies in 2011 were unintended; this is the lowest incidence ever recorded (Guttmacher Institute, 2016c). However, despite this downward trend for women as a whole, still, at the present rate, over half of all women in America will experience an unintended pregnancy by the time they reach age 45 (Guttmacher Institute, 2017).

Importantly, with the overall decline, there remains a significant disparity in unintended pregnancy rates by socioeconomic status (SES), education, race, and ethnicity. As evidence (Finer & Zolna, 2016), in 2011: (a) sixty percent of pregnancies of women with incomes below 100% of the federal poverty level were unintended, as compared to 30% of women with an income over twice the federal poverty level; (b) women who were not high‐school graduates were 1.67 times more likely to have an unintended pregnancy than were women who are college graduates; and (c) every 79 pregnancies per 1,000 non‐Hispanic Black women, 58 pregnancies per 1,000 Hispanic women, and 33 pregnancies per 1,000 non‐Hispanic White women were unintended.

This distribution of unplanned pregnancies, marked by high rates among minority women, especially African Americans, likely reflect the institutionalized and interpersonal discrimination suffered by these groups for hundreds of years (Prather et al., 2018). Pathways from discrimination to unplanned pregnancies include limited access to affordable and effective contraception and a scarcity of reproductive healthcare providers in neighborhoods where high concentrations of minority women live and work (Bailey et al., 2017). Further, limited education, residential segregation, poverty, and having few opportunities for advancement—rooted in social inequalities—also may contribute to a high rate of unplanned pregnancies by influencing decision‐making around sexual behavior, sometimes in efforts to acquire basic needs such as food and shelter (Bailey et al., 2017). Finally, the legacy of medical experimentation and inadequate healthcare has exacerbated a mistrust of the medical establishment, among disenfranchised women (Prather et al., 2018), leading to a bias against contraception that requires doctors’ intervention, and this includes LARC. The overall picture is that women of color, as well as women of other minorities in the United States, often face difficult socioeconomic circumstances which influence their reproductive strategies and choices, including those related to the use of contraception (or not) and, correspondingly, the risk of an unintentional pregnancy.

### 2.3 Risks for women

The consequences of unintended pregnancies for maternal and child health are far‐reaching and can be fatal. In fact, the United States has one of the worst, if not the worst, maternal mortality rates in the developed world. While global maternal death rates have dropped by more than one third from 2000 to 2015, the rate in the United States has more than doubled since 1987. According to the Centers for Disease Control and Prevention (CDC), about 700 women in the United States die each year as a result of complications related to pregnancy or childbirth, and both state and national data have revealed that Black women bear the greatest risk of maternal death (CDC, 2018). According to the CDC, White women accounted for 12.7 deaths per 100,000 live births from 2011 to 2013 whereas African American women accounted for 43.5 deaths per 100,000 live births (CDC, 2018). That means that Black women die from pregnancy‐related issues nearly four times more often than do White women. According to research from the CDC (2018), the most common causes of maternal death in all women are cardiovascular diseases, 15.2%, noncardiovascular diseases, 14.7%, infection or sepsis, 12.8%, hemorrhage, 11.5%, and cardiomyopathy, 10.3%. African Americans have higher rates of some of those diseases than do other racial groups (CDC, 2017), contributed to by economic and social conditions (including racism) that are more common among African Americans than other groups (Healthy People 2020, 2019; see discussion in Geronimus, Hicken, Keene, & Bound, 2006).

Pregnancy also can trigger or exacerbate mental health issues, particularly if the pregnancy was unplanned and unwanted (e.g., Herd, Higgins, Sicinski, & Merkurieva, 2016). In support, a recent meta‐analysis (across 10 studies, mostly from the United States) has estimated the prevalence rate of maternal depression (antenatal to 12 months’ postpartum) to be 21% among women with unplanned (mistimed plus unwanted) pregnancies, which is significantly higher (about twice) than the prevalence of depression among women with planned pregnancies (Abajobir, Maravilla, Alati, & Najman, 2016). These findings are important because maternal depression is associated with mother–child relationship issues and less responsive maternal behavior, and correspondingly predicts developmental issues among infants and children of depressed mothers (review in Field, 2017).

Unplanned pregnancies also can curtail or interfere with women's education, career, and entry or continuation in the labor force (Chiquero, 2010) and therefore chances to attain financial stability. Most policy makers generally operate under the assumption that these effects are particularly detrimental to teenage mothers, although the size of estimated effects (e.g., years in school) varies widely across studies (from no discernible difference between teens vs. older mothers to 2.6 fewer years of education among teen mothers; for review, see Kane et al., 2013). Still, some data have shown that only about 50% of teen mothers receive a high‐school diploma by 22 years of age compared to 89% of young women who had not given birth during their teen years (Perper, Peterson, & Manlove, 2010). In conjunction, teenage mothers also earn less than teens who are not parents (Dahl, 2010) and may need to depend on family and friends or social welfare, which carries stigma and can lead to victimization by society and demands that undermine parenting (e.g., long hours at menial work), and perpetuate poverty.

Four points are important here. First, outcomes associated with unplanned pregnancies of teenagers are likely not the same as outcomes associated with unplanned pregnancies of women who are older and have more resources than most teenagers would be expected to have. Second, there are many factors that make teenage pregnancies a barrier to education; high among them is the lack of awareness about reentry policies among girls, teachers, and school officials that stipulate that young women who are pregnant or mothers can and should go back to school. In addition, they are often deeply affected by financial barriers, a lack of support, and stigma levied by communities and schools alike. Third, estimates of the cost of unplanned pregnancies do not account for the fact that due to discriminatory actions by government, institutions, and citizens, opportunities for productivity and personal growth vary with race, ethnicity, education, and SES whether or not women have had an unplanned pregnancy. Fourth, government reports rely heavily on large‐scale quantitative research that provides a generalized picture devoid of the perspectives of young mothers themselves.

### 2.4 Risks on children

It has been hypothesized that children whose conception was not planned (“unplanned children”) carry a higher probability for developmental problems than do children whose conception was planned (“planned children”). Indeed, studies have shown that during early childhood, unplanned children have poorer physical health (Crissey, 2005) and are more likely to have behavioral issues (Crissey, 2005), as compared to children who were planned; they also seem to have more problems later in life, including aggression (Hayatbakhsh et al., 2011) and mental health issues (David, Dytrych, & Matejcek, 2003), although results are not entirely consistent across studies (Su, 2017).

Processes underlying the developmental issues associated with unplanned pregnancies are undoubtedly complex. Any of the following could be at play because each has been associated with unplanned and particularly unwanted pregnancies as well as with significant developmental problems in young children: (a) adverse birth outcomes (premature birth, low birth weight) (see the meta‐analysis in Hall, Benton, Copas, & Stephenson, 2017), (b) high levels of antenatal stress (Claridge, Lettenberger‐Klein, & VanDonge, 2017), and (c) difficulty in maternal bonding (Barber, Axinn, & Thornton, 1999; Foster et al., 2018; also see Guterman, 2015).

### 2.5 Costs to society

Unintended pregnancies also are public and economic issues. According to a report from the Guttmacher Institute (Sonfield & Kost, 2015), the total government expenditure for unintended pregnancies in 2010 (which included the medical care for women with unintended pregnancies and their infants for 60 months, abortions, and miscarriages) was $21 billion ($14.6 billion in federal expenditures; $6.4 billion in state expenditures). Although astronomic, these estimates could underestimate the cost of an unplanned child because the real cost extends beyond the 60‐month window. Moreover, the estimates do not include costs from pregnancy‐related care paid by other public health programs or other government benefits or the risk of adverse birth outcomes (Hall et al., 2017). According to the same report, gross savings from enabling women to avert all unintended pregnancies in 2010 would have been $15.5 billion.

As mentioned, costs of untended pregnancies also have been described in terms of a loss of human potential/human capital, defined by education, training, and other investments that enhance an individual's productivity and improve economic growth. For society, this predicts a range of labor market challenges such as working days lost (Chiquero, 2010; Johnson & Schoeni, 2011), which impacts aspects of countries’ microeconomics and labor markets and, in turn, countries’ macroeconomics and economic growth (Hsiao & Heller, 2007).

### 2.6 Heterogeneity in “risk”

It is important to underline that heterogeneity in the “risk” associated with unplanned pregnancies, both within and across sectors, is likely related to the kind, magnitude, and number of stressors that women and their family are coping with (Evans & Kim, 2010), as well as the availability of support from family, the community, and state. This means that conclusions of large‐scale population studies on PI may not generalize across all women, and that could be particularly true of those marginalized in and by society. For those women, an unintended pregnancy may not have the same significance as it does for women with more opportunities, less stress, and fewer barriers to a healthy future. In addition, the women themselves may not attribute the same meaning to such a pregnancy. For instance, there is evidence that early childbearing among African Americans in poor urban areas can mitigate consequences of health risks that are faced during adulthood and the risk of being orphaned or widowed (for a review, see Geronimus, 2013).

## 3 LARCs: INTRAUTERINE DEVICES AND IMPLANTS

The high incidence of unintended pregnancies in the United States (and elsewhere) and the potential risks associated with them point to the need for improved access to effective means of family planning, and LARCs are the most effective reversible means of birth control on the market today (American College of Obstetrics and Gynecology, 2017; Trussell, 2011). Brief descriptions of LARCs approved by the FDA are provided next.

### 3.1 Copper IUDs

There are several copper‐based (nonhormonal) IUDs on the worldwide market, but only one is available in the United States (Copper T‐ 380 Paragard Intrauterine Device (TCu380A), Teva Women's Health). The device is T‐shaped, with a stem of polyethylene and two arms encircled by copper wire. It is inserted through the cervix and placed within the uterine cavity. A small thread extends from the device through the cervical canal and into the upper part of the vagina to allow easy removal and regular checking for correct placement. The device is approved to stay in place for up to 10 years; although it may remain effective through 12 (O'Brien, Kulier, Helmerhorst, Usher‐Patel, & d'Arcangues, 2008). The device is considered highly effective, with one study showing that the Paragard “failed” in 0.8 of 100 women during the first year of use (Trussell, 2011).

Pregnancy prevention by copper IUDs is achieved primarily by affecting the sperms’ mobility—effects that are mostly exerted via the devices’ copper ions (Knazická, Lukác, Grén, Formicki, & Massányi, 2012; Ortiz & Croxatto, 2007). Copper IUDs may also work by causing a local chronic inflammatory response in the uterus (Gemzell‐Danielsson, Berger, & Lalitkumar, 2013) and by affecting cell signaling in the sperm, ova, and endometrial lining, thus preventing appropriate implantation. As such, the major action of prevention of pregnancy by copper IUDs occurs prior to implantation (Rivera, Yacobson, & Grimes, 1999), although there are some postfertilization contraceptive effects (Stanford & Mikolajczyk, 2002).

### 3.2 Hormonal IUDs

Four types of hormonal IUDs are on the market in the United States (Mirena, Skyla, Kyleena, and Liletta). All of them have T‐shaped plastic frames with hormonal reservoirs, containing levonorgestrel (LNG), a synthetic progestin that is released through a rate‐controlling membrane (Gold Standard, 2015). The FDA approves the use of the devices for 3 to 5 years, depending on the IUD (Heinemann, Reed, Moehner, & Minh, 2015). The devices are safe, with a failure (conception) rate of 0.2 per 100 women during the first year of use (Trussell, 2011).

Although dosage and duration of contraceptive effects vary across hormonal IUDs, they all work much in the same way, with a mostly local effect at the uterus. The primary mechanism for pregnancy prevention is the thickening of the cervical mucus to stop sperm from swimming up the cervix and fertilizing an egg (Stanford & Mikolajczyk, 2002). The hormone also thins the endometrial lining of the uterus, which limits the ability of a fertilized egg (if one were fertilized) to implant (Sheppard, 1987); this same mechanism causes lighter menstrual periods. The IUD also creates an inhospitable environment in the uterus (Stanford & Mikolajczyk, 2002) and interferes with the cell signaling necessary for implantation (Archer, DeSoto, & Baker, 1999). Hormonal IUDs may also inhibit ovulation, but do not consistently do so (Bayer HealthCare Pharmaceuticals, 2009).

### 3.3 Contraceptive implants

The one implant available in the United States is Nexplanon (Merck & Co., Inc.). The device is a flexible plastic rod about the size of a matchstick that is placed under the skin of the upper arm. The implant contains ethylene‐vinyl acetate copolymer with 68 mg of a synthetic progestin. The hormone is released in a controlled manner over a period of 3 to 4 years (Croxatto, 2002) and is considered the most effective long‐acting reproductive device, with a failure rate of 0.05 per 100 women during the first year of use (Trussell, 2011)

Pregnancy prevention by implants is mediated by the release of etonogestrel, a progestin, at a constant small dose, which inhibits the release of gonadotropins, especially luteinizing hormone, one of the reproductive hormones important in ovulation. It also increases the viscosity of cervical mucus, which hinders the passage of spermatozoa and alters the lining of the uterus to prevent implantation of a fertilized egg into the endometrium (Croxatto, 2002).

## 4 ADVANTAGES, BARRIERS, AND DISADVANTAGES OF LARC

The sharp increase in LARC usage in recent years (Kavanaugh & Jerman, 2018) speaks to promotional efforts and the advantages of LARC as perceived by some women. Still, LARCs are only the third most commonly used type of reversible contraceptives among U.S. women who use contraceptives (26% pill, 15% condom, 12% LARC; Guttmacher Institute, 2016a). This puts women in the United States behind most of the world in usage (United Nations, 2015), and this coalesces with the fact that sterilization is more common in the United States than in many other developed nations (Guttmacher Institute, 2016a). According to a recent, large, representative sample (*N* = 9,321; National Surveys of Family Growth; Kramer, Higgins, Godecker, & Ehrenthal, 2018), LARC use differs significantly by race and ethnicity: Nine percent of White women, 11% of Hispanic women, and 7% of Black women reported currently using LARC (2011–2013 and 2013–2015 pooled; *P* = .03). Next, we discuss advantages and disadvantages (negative aspects of LARC), heterogeneity in women's perception of what is an advantage or disadvantage based on personal considerations, and finally, a description of the “external” (policy) barriers that make access to information about LARC or to the devices themselves difficult for some women.

### 4.1 Advantages

LARCs have several advantages over other methods of birth control, including the fact that they are more effective in preventing pregnancy than are birth control pills or condoms (American College of Obstetrics and Gynecology, 2017; Trussell, 2011). In fact, the efficacy of LARCs is about equal to that of tubal sterilization, but has the advantage of being reversible (Trussell, 2011). The efficacy of LARCs also corresponds with a low rate of ectopic pregnancies, although if a woman does become pregnant with an IUD in place, she is at increased risk for an ectopic pregnancy as compared to women using other methods of birth control (Vessey, 2016). Another advantage is the convenience of LARCs because they essentially can be forgotten between medical visits unless users suffer from side effects (e.g., change in bleeding pattern, cramping, bleeding; see reviews in Curtis & Peipert, 2017 and Strasser, Borkofski, Couillard, Allina, & Wood, 2016). In addition, LARCs are estrogen‐free and therefore do not carry the same significant (although low) risks for thrombosis as do some other estrogen‐containing contraceptive techniques (Charlton et al., 2014). This is especially important for women with a clotting tendency (thrombophilia). We also note that although LARCs have high upfront costs, they can remain in place for up to 12 years, so they may be cheaper overall. According to one cost analysis, LARCs are cost‐effective if in place at least 2 years (Trussell et al., 2013). Finally, some women with LARCs report greater sexual enjoyment because they can be spontaneous during sex and still be confident of pregnancy protection (Higgins, Sanders, Palta, & Turok, 2016). Likely for these reasons, women tend to think highly of IUDs and implants, as demonstrated by reports of high satisfaction and continuation rates (Peipert et al., 2011).

### 4.2 Disadvantages of LARCs

Among the disadvantages of LARCs is that insertion is uncomfortable and sometimes painful, particularly among nulliparous women (Foran, Butcher, Kovacs, Bateson, & O'Connor, 2018), and the most effective method of pain control has not yet been established (see review in American College of Obstetrics and Gynecology, May 2018, pp. e134–135). LARCs also may cause side effects such as menstrual pain and bleeding, spotting, headaches, nausea, and mood changes. In addition, the high cost of LARCs (depending on insurance coverage) and the need to see a doctor to insert and remove the devices may be off‐putting to some women, especially if they are planning to have a baby in the near future. Some women want to avoid insertion of a foreign body into their body or devices that can be detected by a partner during intercourse. LARCs also do not protect women against sexually transmitted diseases (STDs), so that in circumstances in which protection is wanted, a condom or other barrier contraception needs to be used along with a LARC. This limits the convenience of LARCs and adds to the cost of contraception if it is not paid by insurance. In addition, not all women can safely use LARCs, particularly those with a current pelvic infection or an STD, gynecologic cancers, or other serious illnesses (Curtis & Peipert, 2017). Finally, women may not choose LARCs due to personal experiences or the experiences of others, the relationship context, or ambivalence about wanting to conceive (for a review, see Gomez et al., 2014).

### 4.3 Heterogeneity in women's perceptions of advantages/disadvantages

At this junction, it is important to state that what some women see as an advantage of using LARCs may be regarded as a disadvantage by other women (Kavanaugh, Frohwirth, Jerman, Popkin, & Ethier, 2013). For example, the long‐lasting nature of IUD protection is regarded as a positive for women who want to delay childbearing for a number of years, but as a negative for women who are uncomfortable with the idea of long‐term use. Similarly, menstrual suppression associated with a hormonal IUD can be regarded as favorable or as a downside; for example, some Latinas who have cited cultural beliefs, despite data to the contrary, about the harmful effects of not getting a regular period (White, Hopkins, Potter, & Grossman, 2013). Finally, the necessity of having LARCs inserted and removed by a doctor appeals to some respondents because it takes control out of their hands, yet others see the lack of control as a disadvantage.

### 4.4 Barriers to use

There are many “external” barriers related to policy and healthcare practices that can stand in the way of women using LARC, and we divide them into three subsections: client‐level, provider‐/clinic‐level, and system‐level barriers, although the categories are somewhat intertwined.

#### 4.4.1 Client‐level

A primary barrier for women is the high up‐front cost of LARCs (Foster et al., 2015). Planned Parenthood estimates the total cost (i.e., for insertion, cost of device, and follow‐up visit) of an implant in the United States as $400 to 1,000, and for an IUD, $500 to 1,000 depending on the device (Eisenberg, McNicholas, & Peipert, 2013). Notably, under the Patient Protection and Affordable Care Act, 2010 (Affordable Care Act, ACA, also known as “Obamacare”), the devices are fully covered, although with some stipulations and variations in cost by state, region, and clinical setting (Sonfield, 2015).

Other issues include (a) the need to go to a provider for insertion and removal of a LARC, which can be costly in terms of childcare, transportation, and lost days at work, which would be especially burdensome for low‐income and poor women; (b) the idea of depending on “another” to start and end contraception can be very scary to some women, particularly those with a historical legacy of reproductive injustices that have and continue to be imposed on them (discussed later); and (c) delayed or disallowed access by providers who incorrectly believe that LARCs cannot be used after childbirth, in nulliparous women, or after an abortion (e.g., Foran et al., 2018).

An additional client barrier is mostly specific to minors and regards the issue of consent and confidentiality (American College of Obstetrics and Gynecology, May 2018). Although federal regulations state that minors can seek contraceptive care from federal sources without parental consent, issues of consent for adolescents covered by private insurance are governed by state laws, which vary considerably. Twenty‐one states have explicitly stipulated that adolescents may seek family planning services without parental consent, 25 states allow it under certain circumstances, and 4 states have no specific policy regarding this issue (Guttmacher Institute, 2016b). Further, even if adolescents are able, independently, to consent to contraceptive care, this does not necessarily guarantee confidentiality (see Kumar & Brown, 2016). For instance, some billing practices (e.g., explanation‐of‐benefits notifications) can compromise confidentiality, especially if minors are using parents’ insurance benefits to pay for contraception (Andrasfay, 2018).

#### 4.4.2 Provider barriers

An often cited barrier to LARC usage is the lack of providers’ knowledge about the safety and effectiveness of LARCs and insufficient training in insertion and removal of the devices (Harper et al., 2012; Harper et al., 2013). In surveys, medical providers have described reservations in providing LARCs because of a risk of infection and liability (Harper et al., 2008), despite the good safety record of today's devices. As mentioned, some providers still believe that LARCs should not be used by women who have never given birth or by women immediately after birth or after an abortion, which also is not the case (American College of Obstetrics and Gynecology, 2017; Foran et al., 2018). A lack of LARC education among providers is particularly salient in federally qualified health centers that serve many women of low SES (Beeson et al., 2014).

Even providers who are familiar with and favor the use of LARCs do not always offer them in practice (Luchowski et al., 2014). In one focus group, some providers had voiced the belief that patients and not providers are responsible for initiating discussions about contraceptives (Akers, Gold, Borrero, Santucci, & Schwarz, 2010).

At the other extreme, there have been reports by women describing a provider's pressure on them to adopt a LARC, even if the clients preferred another means of contraception, and this seems to be most prevalent during counseling of women who are marginalized, particularly those who are Black and poor (Higgins, Kramer, & Ryder, 2016). Along the same lines, women have reported that providers minimize side effects of LARCs or disregard women's request to remove theirs unless they are very insistent (Higgins, Sanders et al., 2016). These behaviors on the part of providers can make women feel disrespected or patronized during provider–patient interactions regarding contraception. Such experiences can undermine patients’ trust in their provider and raise doubts as to his or her recommendations.

#### 4.4.3 System‐based barriers

Some of the current healthcare policies make it difficult to know about and access LARC, particularly among women who are without financial resources. One example is the complicated set of policies involved in providing access to contraception and services to women who lack coverage from an employer‐provided health insurance plan. For these women, benefits come from either the ACA or providers receiving Title X funding.^2^ However, for women who lack these sources of coverage, contraceptive counseling and services are available free from providers funded by Title X if the women's income falls below the federal poverty guidelines; if above that, the cost is calculated according to a sliding scale or charged in full. These policies can make it difficult to navigate the system in which some women pay and some do not and with availability to some women limited to specific providers (Wu & Mark, 2018).

Other policy issues are: (a) Double‐billing IUDs, once for insertion and one for removal. As a result of this policy, women who lack insurance or have inconsistent coverage may fear or face financial barriers for removal and consequently opt for cheaper contraceptive options that that can be discontinued by themselves. (b) The still‐inadequate mechanisms of reimbursement for inpatient LARC insertion during hospitalization, after childbirth, means that women have to delay use of LARC contraception after giving birth and make a special visit to a provider for insertion, which could be difficult for women who are poor and/or live in areas without a provider close by (Rodriguez, Evans, & Espey, 2014). (c) Publicly funded centers may not stock LARCs because of their high cost, thus making access difficult for low‐income women who use the clinics (Beeson et al., 2014). These barriers are the highest and most impermeable to the 60% of noncitizen immigrant women in the United States (twice the percent of citizens) who are uninsured (Planned Parenthood Action Fund, 2015). The high price tag of LARCs probably puts them out of reach for most of these women.

Finally, Catholic hospitals operate according to the Ethical and Religious Directives for Catholic Healthcare Services (ERD), guidelines for healthcare delivery issued by the United States Conference of Catholic Bishops (2009). Thus, healthcare at these hospitals must follow Catholic moral teachings, and the ERDs prohibit access to common reproductive services such as contraception and abortion. In these regards, an especially hard line is taken against IUDs because they may prevent a fertilized egg from implanting and therefore are considered abortions or “abortifacients,” which are forbidden by Catholic doctrine (deBlois & O'Rourke, 1995). Some Catholic lobbyists also have raised the issue of potential medical complications associated with the use of IUDs and argue that the “temporary sterilization of women and girls” (by LARCs) does nothing to prevent STDs and does not address the psychological and medical risks and costs associated with increased sexual activity (LaPoint, 2015; for a review, see Catholics for Choice, 2017).

On these bases, the Catholic Church lobbies strongly against subsidies for LARCs and puts up multiple barriers for contraception provision (Liu, Hebert, Hasselbacher, & Stulberg, 2018). These can include variable institutional policies and enforcement of contraception restrictions, word‐of‐mouth admonishments, and lease agreements prohibiting contraception in secular clinics on Catholic‐owned land. Notably, in January 2018, the Department of Health and Human Services announced the creation of a Conscience and Religious Freedom Division within its Office of Civil Rights. The purpose of the new division is to better enforce 25 existing federal statutes that allow healthcare workers to refuse to provide care that they believe conflicts with their religious beliefs or moral convictions.

These policies jeopardize access to LARCs and restrict some other healthcare services as well. This puts providers working within Catholic systems, who do not share their employers’ religious objection and believe that they have a duty to provide care, in a difficult position (Liu et al., 2018). Sometimes, these providers can work around the restrictions by referring patients to providers in nonaffiliated or secular clinics or hospitals. However, this can burden patients, especially from underserved populations, and often results in delayed or lower quality care. Further, many women may not know that they are being served by a religious hospital and enter the facility without knowing that it does not provide all forms of care (Wascher, Hebert, Freedman, & Stulberg, 2018). After discovery, these women have to find another facility and provider, which may not be easy (e.g., if the only facility in the area where they live is religious) or timely.

## 5 LARC: EFFORTS TO INCREASE USE

Because of LARC's effectiveness, initiatives have been designed and implemented to encourage use. A prime example is the Contraceptive CHOICE project, which provided counseling and no‐cost contraception to women in the St. Louis, Missouri area in an effort to curtail unintended pregnancies (Birgisson, Zhao, Secura, Madden, & Peipert, 2015; Broughton et al., 2016). Of the nearly 10,000 women counseled on all types of birth control using a tiered approach (with the most effective methods described first; Stanback, Steiner, Dorflinger, Solo, & Cates, 2015), 75% chose a LARC as their method of contraception. This rate is similar to that reported by Madden et al., 2018, who reported a 76.1% uptake of LARCs among a sample of CHOICE‐participants compared to 4.8% among a “simulated” comparison group. Estimates of this latter group were based on data obtained from the Missouri Title X program and adjusted for relevant covariates.

Follow‐up data from CHOICE‐participants show that LARC users were 22 times less likely to experience an unplanned pregnancy, and abortion rates for CHOICE‐participants were less than half of those living in the surrounding region (and therefore not eligible for the CHOICE project) (Birgisson et al., 2015). The program had the greatest impact on teen pregnancies, births, and abortion rates, which declined more rapidly than the national average (Birgisson et al., 2015). According to a recent cost analysis (Madden et al., 2018), the total cost savings for the state of Missouri attributable to the Contraceptive CHOICE Project was estimated to be $5.0 million over the project duration.

These results should be regarded with some caution. They may not be entirely generalizable to the larger population because participants were recruited from specific sites that provided contraceptive counseling and services to low‐income women, and participants came to the sites seeking contraception. In addition, frontline providers may have guided participants to LARCs over other methods, given the goals and design of the project (Stanback et al., 2015). Women in the study also were not randomly assigned into control and treatment groups. For these reasons, the study cannot provide a firm answer as to what LARC take‐up would be if the devices were free and universally accessible and offered along with other devices with ample information on each one (for a full discussion, see Madden et al., 2018).

One other large‐scale initiative to increase LARC uptake is the $23 million Colorado Family Planning Initiative (CFPI; Ricketts, Klingler, & Schwalberg, 2014) aimed at increasing the use of LARCs through counseling and the lowering or covering of their costs in Title X clinics. Results of recent analyses have attributed a 6.4% decrease in teen birth rates over 5 years to the project, with higher estimates (10.4%) in counties with poverty rates above Colorado's median (Lindo & Packham, 2017). These findings translate into an overall estimate of about 6,000 LARCs provided to teenagers through the CFPI initiative and approximately 1,500 teenage births prevented between 2009 and 2013. This relatively large effect has suggested that the program had success; however, the findings are limited to teen births and so cannot be generalized to other populations.

Another type of initiative is aimed at facilitating policy: adherence and implementation. One example is the founding of the Immediate Postpartum LARC Learning Community—a cross‐state collaboration—to facilitate information‐sharing and support states, with the end goal of improving access to LARCs immediately after birth through policy implementation. The initiative can establish best practices and strategies for effective service delivery and serve as a mediator of system change (Kroelinger et al., 2018).

In addition to these and other large‐scale projects, there are other smaller initiatives in place. Examples are projects that center on online and telephone tools to complement in‐person counseling on LARCs (Gilliam, Martins, Bartlett, Mistretta, & Holl, 2014; Sridhar, Chen, Forbes, & Glik, 2015), projects to increase provider knowledge, and trials to test the efficacy of providing access to LARCs immediately after an abortion in order to prevent repeated pregnancies (Langston, Joslin‐Roher, & Westhoff, 2014).

## 6 LARC AS ROUTES TO SOCIAL INJUSTICE

The initiatives to promote LARC are in keeping with the national health goal of reducing the number of unintended pregnancies in the United States (Healthy People 2020, 2011). On one hand, this can be justified as favoring the public good, assuming that such efforts promote the well‐being of current and future generations. On the other hand, childbearing, pregnancies, contraception, and sexual activity are private behaviors and involve profoundly private decisions that society should leave for individuals to decide for themselves.

In this context, it is critical to acknowledge a historical legacy, perpetrated by calculated acts of White supremacy, colonialism, classism, able‐ism, and misogyny that pushed for and legislated policies that were said to be aimed at the public good, but overrode the rights of some individuals, particularly the most vulnerable women, to curb or stop their reproduction. Examples of past abuses include the control of Black women's fertility and sterilization during slavery; of Native Americans during the settlement of the United States; and of poor people, immigrants, prisoners, and men and women hospitalized in psychiatric facilities in response to the American eugenics movement (late 1800s–1940s) that sought to increase births among the “fittest” and limit births among the “unfit” (Currell, 2006).

Other, more recent examples include the arrests of and forced interventions (e.g., cesarean sections) on women whose pregnancy was a necessary factor leading to attempted and actual deprivations of their physical liberty. Paltrow and Flavin (2013) described 413 such cases from 1973 to 2005. According to records, the basis for the arrests and/or interventions in many of the cases was related to “saving” the fetus, mostly under extremely dubious circumstances such as wanting to have a vaginal birth after having a cesarean section or due to a miscarriage or still birth that was blamed on the mother. Although Paltrow and Flavin noted that such intrusive practices have occurred in every region of the country and affect women in all sectors and strata (p. 333), they also described disturbing disparities by race, ethnicity, and SES, with poor and/or African American women (especially from Southern states) being particularly vulnerable to many of the insults (Paltrow & Flavin, 2013).

Injustice toward women's reproductive rights, especially among women who are socially marginalized, continues today. Examples pertaining to LARC include recent policies adopted by a number of U.S. states that create incentives or disincentives to limit childbearing by women receiving public assistance (for a full discussion, see Strasser et al., 2016). Other examples of discrimination include biased renditions of contraceptive options offered to some women, particularly women of color and poor women (Dehlendorf et al., 2010). Subtle discriminatory biases also are seen in a lack of funding in some state Medicaid programs for device removal if the recipient intends to get pregnant (South Dakota Department of Social Services, 2016).

These past and present policies serve as examples of discriminative acts against marginalized populations of women by directing them toward a particular contraceptive and reproductive “strategy.” Such policies and practices can cause women to distrust the “system” and their provider and can elicit fears of being forced to use a particular contraceptive strategy without their permission and to their physical detriment. Ethically, such targeting practices negate the rights of women to choose the kind of contraception that they want to use. In this context, advocates of RJ call for putting the priorities, needs, and preferences of individual women—not the promotion of specific technologies—first.

To attain this means disallowing practices among clinicians, providers, and policy makers that impose a directive view of “what is best” for women and society by promoting one method of contraception over others. It also entails eradicating barriers to all medically approved contraception so that they are equally available to women, regardless of sociodemographics. It means assuring health literacy and leaving the choice to women to decide without pressure or skewed advice about the full range of contraceptives that are available. As described by Dehlendorf, Krajewski, and Borrero (2014),

Contraceptive counseling should take the form of “a shared decision making approach [between provider and client] that focuses on eliciting and responding to patient preferences and [uses] specific task‐oriented communication strategies to enhance the process of method selection, facilitate correct use of a chosen method, and meet women's overall reproductive health needs.” (pp. 669–670)

As well, policies that impinge on women's freedom to use the contraceptive method of their choice must be expunged and rewritten to allow all women full reproductive rights. In these ways, women across the sociodemographic continuum would be offered contraception with the respect and freedom that they deserve.

## 7 PRO‐CHOICE VERSUS RJ

Ensuring the rights of all women to choose the contraception that they deem appropriate is an important goal. However, proponents of RJ have argued that assuring choice is not enough because decisions about reproduction are very complicated, and the right to make a choice does not help a woman figure out what “choice” means in her individual context and what the best choice might be. For this, women may need guidance, information, real options, and meaningful support to mindfully make a decision. Practically, this calls for more education, access to a good health provider, health insurance, resources, transportation, the ability to take time off from work to get to an appointment, childcare during a medical visit, and documentation of immigration status. Above all, RJ calls for ensuring that people have full bodily autonomy, have the rights to reproductive choice, and are provided with the chance for them and their children to live healthy lives with promising futures. The movement also calls for the persons most affected by discriminative policies and practices to serve as leaders in shaping the collective analysis and by leading efforts toward reimagining and building just communities and societies. As such, it emphasizes social justice, which removes reproductive decisions from an individualized space and makes it part of a broader set of community and national priorities (Ross, 2006/2011).

From this perspective, whether a woman can or should use LARCs is really not *the* issue, although they are an effective means of curtailing unwanted pregnancies. Rather, *the* central issue is the challenges facing poor women, women of color, and in fact, to some extent, all women, grounded in the socioeconomic and cultural biases perpetuated in a society that allow for some persons to attain individualized goals more than do others. Seen in this way, the remedy is no less than to make social equity a primary priority everywhere and for everyone. According to advocates, although unbiased consultations and free and easy access to LARCs could be significant advances toward this goal, they are seen as only relatively small steps on the road to social and RJ.

## 8 RELEVANCE TO IMH PROFESSIONALS

IMH professionals should learn about and contribute to these efforts because RJ affects the well‐being and future of parents, families, and children, who are the focus and aim of work in the field of IMH. For professionals, as individuals, learning about RJ can help them develop a more critical awareness regarding their own social identities, consider how these identities situate them within hierarchical social systems of privilege and oppression, and examine how their identities impact their attitudes and care of families. Learning about RJ can also prompt IMH professionals to learn more about multicultural issues that promote a better appreciation of the impact that past and present social conditions (including all kinds of oppression and discrimination) can have on individuals, their health, personal/family relationships, and the manner in which they negotiate their world.

IMH professionals also can contribute a great deal to the RJ movement via their research, clinical practice, teaching, and advocacy. As a few examples of IMH research that could contribute to RJ: (a) Studies that examine whether LARC use, indeed, improves women's and children's lives (e.g., mental health) in the long‐term, and if so, whose lives and in what ways; (b) research with an end goal of designing and evaluating tools and interventions to increase the resilience of adults and children in the face of discrimination by institutions or individuals in efforts to empower those who are discriminated against and help them cope and protect themselves, and (if age‐appropriate) to encourage action toward progress and reform; (c) qualitative research that offers an understanding of the perspectives of women from different sectors regarding reproductive health and of the broader contextual forces that contribute to their contraceptive choice; and (d) studies that provide insight on when and how to talk to children about discrimination, racism, and personal rights, including those related to gender and reproduction. As a final suggestion, IMH researchers could offer a further understanding into the meaning of “racism” as it is displayed and transmitted in contemporary society, now that some aspects of overt racism have been made illegal (Bradby, 2010). A failure to update our thinking in this regard will undermine efforts to address the complexity of racism and the many ways that it influences care providers and clients as well as their perceptions of one another and their interpersonal communication (Peek et al., 2010).

In the clinic, practitioners who are knowledgeable and sensitive to issues related to RJ and social justice may better appreciate the diversity of women's stories. They also may be better able to help clients (adults and children) cope with past personal traumas related to reproductive and social injustices. In this way, clinicians can help clients work toward resolution so that going forward, these individuals can be healthier and, if they are parents, can raise their children without the negative impact of stress and trauma on body, brain, and behavior (Geronimus et al., 2006).

As teachers, IMH professionals can integrate social justice theory into training programs and clinical supervision of students (e.g., see Mallinckrodt, Miles, & Levy, 2014). In this regard, it will be important to go beyond cultural and linguistic competence, which is mandated in some clinics and medical schools, to address larger questions of systemic bias (Metzl, Petty, & Olowojoba, 2018). Attainment of competency in these matters (called *structural competency*) calls on mental health care providers and students to recognize the ways in which institutions, markets, and healthcare delivery systems shape symptom presentations, clinician–client communication, and healthcare disparities.

As advocates, IMH professionals can help destigmatize contraception and abortion through open discussions with clients and inform them, the public, and policy makers of the many ways in which RJ affects family and children. More broadly, putting forth the conceptualization of the child, as seen within the IMH framework, could serve the discourse on RJ and social justice by offering a nuanced understanding of the diversity of children's experiences and the routes by which diseases and health are exacerbated by social, economic, environmental, and political milieus.

In all of these ways, specialists in IMH can stand in solidarity with advocates of RJ and contribute to the building of a society where *all* women have the right to health literacy, personal bodily autonomy, have children or not have children, and parent their children in safe and sustainable communities. By advancing toward this goal—together—there will be louder voices, more advances, and greater hope that all women and their families and children will be afforded the best possible conditions and opportunities to grow, develop, and prosper.

## CONFLICT OF INTEREST

The authors report no conflict of interest.

## ENDNOTES

1 Medicare is a joint federal–state program that provides low‐cost or free medical coverage to persons and families with low‐income, pregnant women, the elderly, and people with disabilities.

2 Title X is a federal grant program dedicated to providing individuals with comprehensive family planning and related preventive health services. Title X is legally designed to prioritize the needs of low‐income families or uninsured people who might not otherwise have access to healthcare services. These services are provided at reduced or no cost.
